# Learning curve for mastery of colorectal endoscopic submucosal dissection: Perspectives from a large Japanese cohort

**DOI:** 10.1002/jgh3.12298

**Published:** 2020-01-15

**Authors:** Leonardo Zorron Cheng Tao Pu, Takeshi Yamamura, Masanao Nakamura, Masaya Esaki, Uayporn Kaosombatwattana, Miguel R Rodriguez, Suzanne Edwards, Alastair D Burt, Rajvinder Singh, Yoshiki Hirooka, Mitsuhiro Fujishiro

**Affiliations:** ^1^ Faculty of Health and Medical Sciences The University of Adelaide Adelaide South Australia Australia; ^2^ Department of Gastroenterology and Hepatology Nagoya University Nagoya Japan; ^3^ Department of Endoscopy Nagoya University Hospital Nagoya Japan; ^4^ Department of Medicine Siriraj Hospital, Mahidol University Bangkok Thailand; ^5^ Department of Liver, Biliary Tract and Pancreas Diseases Fujita Health University Toyoake Japan; ^6^ Department of Gastroenterology Lyell McEwin Hospital Adelaide South Australia Australia

**Keywords:** colorectal neoplasms, efficacy, endoscopic submucosal dissection, learning curve, safety

## Abstract

**Background and Aim:**

Endoscopic submucosal dissection (ESD) is a challenging procedure. A dissection speed of ≥9 cm^2^/h has been acknowledged as a mark for expertise, alongside a complication rate of ≤5% and en bloc resection rate of ≥90%. However, there is lack of objective information on whether the three measures correlate with each other. This study aims to evaluate the dissection speed, safety, and efficacy of colorectal ESDs performed by experts and trainees.

**Methods:**

Consecutive patients undergoing colorectal ESD at a Japanese hospital (2006–2017) were included in a prospectively collected database. Information on patient demographics, proceduralist, and intra‐/postprocedure data was retrieved. The primary outcome was the comparison in dissection speed. The secondary outcomes included differences in safety and efficacy. Log‐linear regression models adjusted for confounders (e.g. R0 resection) were used to assess the differences in dissection speed.

**Results:**

Five hundred ninety procedures (514 patients) performed by 26 endoscopists were analyzed. Experts performed a higher number of difficult lesions (e.g. F2 fibrosis) but achieved higher dissection speed (10.3 *vs* 6.7 cm^2^/h). The difference was statistically significant for both unadjusted and adjusted models (*P* < 0.0001). The en bloc resection rates were similar for both groups (experts = 95.6%; trainees = 94.7%, *P* = 0.61). Although nonexperts damaged more of the muscularis propria (18.6 *vs* 12.5%, *P* = 0.04), this did not translate into a significant difference in perforation (experts = 3.7%; trainees = 6.9%, *P* = 0.09) or delayed bleeding (experts = 2.9%; trainees = 4.4%, *P* = 0.34). The dissection speed steadily increased with expertise.

**Conclusion:**

Although dissection speed for colorectal ESD was significantly higher for experts, ESDs could be safely and efficaciously performed by ESD trainees.

## Graphical abstract

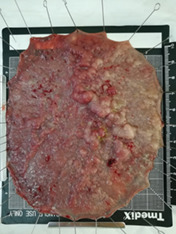

Endoscopic submucosal dissection (ESD) expertise is commonly measured through dissection speed, en bloc resection, and complication rates. However, it is unclear whether the slower pace during ESD training is necessarily accompanied by worst outcomes regarding efficacy and safety. This study found that colorectal ESD can be safely and efficaciously performed by ESD trainees.

## Introduction

Endoscopic resection is the current gold standard for treatment of precancerous and early cancerous lesions within the gastrointestinal tract. Different modalities of endoscopic resection have been proposed and the decision of one over the other depends on several factors, one being the degree of dysplasia/invasiveness of the lesion. For lesions involving superficial submucosa, guidelines from both West and East advise on the use of endoscopic submucosal dissection (ESD).^1^, ^2^, ^3^, ^4^

There are different learning curves depending on endoscopist factors such as prior experience with other therapeutic endoscopy procedures (e.g. endoscopic mucosal resection [EMR]) and experience in assisting ESDs.^5^ Different learning curves depend on the endoscopist's expertise and the location of the ESD within the gastrointestinal tract. As ESDs performed in the stomach are easier to tackle than in the esophagus and rectum/colon, it is advised to use gastric ESDs first for ESD training.^6^ This is feasible in Asian countries owing to their relatively high prevalence of early gastric cancer. However, in Western countries, this is not a reality, and hence the choice for training falls into the lower gastrointestinal tract.

In selected scenarios, a few dozens of ESDs performed in humans suffice for achieving proficiency. Fewer cases are thought to be necessary if animal models are used for training before performing in humans.^7^ However, this has been mainly postulated based on gastric ESDs. Although there are studies looking into colorectal ESD learning curves in both the West and East, they mainly focus on single‐operator experiences.^8^, ^9^, ^10^

A definitive number of procedures to achieve proficiency in ESD is difficult to determine. This is not only due to variation in personal skills, but also due to the lack of objective standardized markers for expertise. In order to determine the minimum standards for ESD skills, a group of experts gathered evidence from multiple studies and advised thresholds for “ESD proficiency,” mainly based on three variables. According to Oyama *et al*., for an endoscopist to be considered skilled in ESD, he or she should achieve: (i) dissection speed ≥9 cm^2^/h; (ii) complication rate ≤5%; and (iii) en bloc resection rate ≥90%.^11^ However, objective information on how these three measures behave throughout the ESD learning curve is scarce. In this study, we intend to evaluate the learning curve of a Japanese endoscopist cohort in gaining proficiency toward colorectal ESD. As proposed by Oyama *et al*., we specifically investigated the evolution of dissection speed, safety, and efficacy throughout the process.

## Methods

We retrospectively assessed the Nagoya University Hospital's prospective database of colorectal ESDs and included all the patients who were submitted to colorectal ESDs from 2006 to 2017. The final decision of proceeding with the ESD was made based on endoscopic imaging after topical administration of 0.4% indigo carmine, virtual chromoendoscopy (either narrow‐band imaging [NBI] or blue‐laser imaging [BLI]), and crystal violet at 0.05%. From this initial cohort, only the endoscopists who had performed more than one ESD during his or her attachment to the department were included in the study.

We divided colorectal ESDs into two groups according to the executor: ESD trainee group and ESD expert group. Each ESD trainee performed on average 16 colorectal ESDs in our center before finishing the colorectal ESD supervised training program. Subjectively, the trainees who completed their training were considered proficient in our center. Therefore, the ESD expert group consisted of those in the ESD trainee group who continued with their attachment to the department after their 16th colorectal ESD. In other words, if an endoscopist had performed 20 colorectal ESDs while at Nagoya University Hospital, the data on the first 16 ESDs were used for the ESD trainee group and the last 4 were used for the ESD expert group. All endoscopists included in the ESD expert group had their initial 16 colorectal ESDs included in the ESD trainee group.

**Figure 1:**
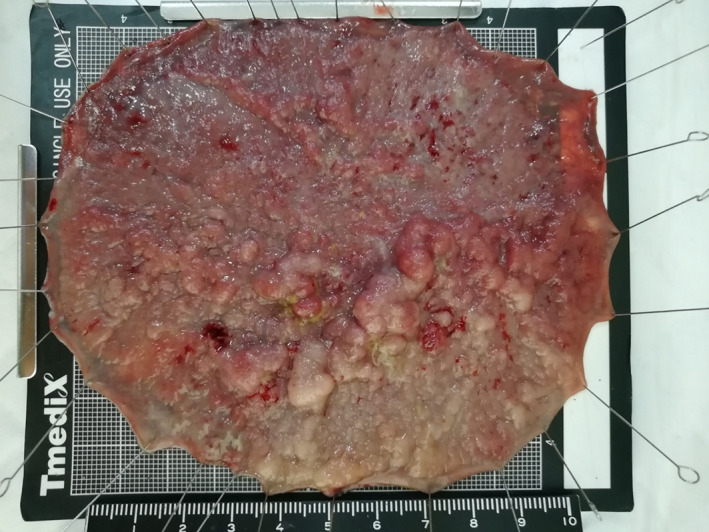
Example of endoscopic submucosal dissection specimen measurement.

We retrieved information on patient demographics, lesion endoscopy and pathology features, and procedure details (e.g. time for completion). The size of each lesion was measured with a ruler after resection and fixation onto a plate (Fig. 1—Example of ESD specimen measurement). Lesion areas were calculated according to their shape (circular or oval) and based on the two major measured diameters after resection (π × length × width/4), and expressed in cm^2^. The area was then divided by the procedure time in hours for determining the average dissection speed in cm^2^/h. Procedure time was defined as the time from first incision until the retrieval of the specimen (including the time for management of complications). Fibrosis was expressed in three categories (i.e. F0‐2) as previously described.^12^ Complications included intraprocedure muscularis propria (MP) damage (excluding perforation), perforation, post‐colorectal ESD coagulation syndrome (PECS), delayed bleeding (that required endoscopy or surgery after ESD), abdominal pain (promptly after the procedure), and fever (temperature above 37.5°C). Curative resection was defined as ESD R0 procedures for patients with lesions up to 1000 μm into the submucosa. PECS was defined as abdominal pain without perforation as per Arimoto *et al*.^13^

The primary outcome was to analyze the differences in average dissection speed between colorectal ESD trainees and experts. The secondary outcomes included the differences in safety and efficacy between the two groups.

Procedures were performed after split bowel preparation, using conscious sedation and carbon dioxide insufflation. The injectate fluid was prepared with hyaluronic acid and saline in a 1:1 proportion and had adrenaline in a 1:200 000 dilution. A small amount of indigo carmine (~1 mL/200 mL) was also added to the solution. The main knife used for dissection was Flush Knife BT‐S 2.0 (©Fujifilm Corporation, Tokyo, Japan) and that used for bleeding control was Coagrasper (©Olympus, Tokyo, Japan). This was connected to a water jet pump (Fujifilm JW‐2 or Olympus OFP‐2) with saline dyed lightly blue by indigo carmine. The video endoscopes used were either from Fujifilm (600 series) or Olympus (260 series) and as a rule consisted of pediatric colonoscopes for lesions in the right colon and gastroscopes for lesions in the left colon. All ESDs involved the use of disposable distal hoods (©TOP Corporation M‐02, Tokyo, Japan). For electrical cutting and coagulation, VIO ICC 200, 300 D, or 3GI was used as the power source (ERBE Elektromedizin, Tübingen, Germany). The standard settings for ESD in our center are Endocut I effect 2; forced coagulation effect 2, 40 W; and soft coagulation effect 5, 60  W. For adverse event monitoring, all patients stayed in the hospital for 1 week after the colorectal ESD. Specialist gastrointestinal pathologists assessed the ESD specimen in all cases.

Log‐linear regression models adjusted for a priori confounders were used for determining differences in dissection speed. Confounders included difficulty (fibrosis score, lifting sign, ileocecal [IC] valve or anus involvement, lesion beyond fold, retrograde position use, and lesion size), safety (MP damage, any complication, delayed bleeding, perforation, PECS, and emergency operation), and efficacy variables (R0 resection, curative resection, and en bloc resection). Chi‐squared tests were used to assess the differences between proportions, and Student's *t*‐tests were used to assess differences between means using the MedCalc calculator (©2019 MedCalc Software bvba, Ostend, Belgium). The statistical software used to perform adjusted log‐linear models was SAS 9.4 (SAS Institute Inc., Cary, NC, USA). *P* values  < 0.05 were considered significant. This study was approved by the Nagoya University Hospital Human Ethics Review Committee under the number 2015‐0485. All data were coded, and patient anonymity was guaranteed for all nonessential/nonmedical personnel.

This study has been approved by the Ethics Review Committee from Nagoya University Hospital.

## Results

Six hundred fifteen ESD procedures performed in 529 patients between 2006 and 2017 were initially assessed. Thirty‐six endoscopists participated in these procedures. Twenty‐five ESDs from 15 patients performed by 10 endoscopists were excluded from the analysis (endoscopist with only one ESD or procedure aborted after advanced imaging). The final dataset of 590 procedures from 514 patients performed by 26 endoscopists were analyzed. Two hundred seventy‐two (46.1%) procedures were performed by the expert group. The mean patient age was 67.8 (SD = 11.2) and 357 (60.5%) were male. The average major diameter of the specimen was 3.5 cm (SD = 1.8) and the average area was 13.7 cm^2^ (SD = 13.9). An average of 110 min (SD = 72.7) was required to complete the ESD. The descriptive statistics for ESD trainees and experts are summarized in Table 1.

**Table 1:** Cohort characteristics per group

*n* (%)	Experts	ESD trainees	*P*‐value
Number of ESDs, *n* (%)	272 (100)	318 (100)	NA
Male, *n* (%)	169 (62.1)	188 (59.1)	0.46
Age in years, average (SD)	67.8 (10.8)	67.8 (11.5)	0.97
Right colon location, *n* (%)	97 (35.7)	113 (35.5)	0.96
Rectum location, *n* (%)	87 (32.0)	114 (35.8)	0.33
Adenomas, *n* (%)	58 (21.3)	56 (17.6)	0.26
M adenocarcinomas, *n* (%)	149 (54.8)	176 (55.3)	0.90
Superficial adenocarcinomas†, *n* (%)	29 (14.9)	25 (11.5)	0.22
Invasive adenocarcinomas†, *n* (%)	16 (8.2)	17 (7.8)	0.86
Carcinoid, *n* (%)*	3 (1.1)	12 (3.8)	0.04
Other, *n* (%)	17 (6.3)	32 (10.1)	0.10

* *P* < 0.05.

† Threshold of 1000 micrometers into the submucosa.

N/A, not applicable; ESD, endoscopic submucosal dissection.

Of the 26 ESD trainees, 13 had performed more than 16 ESDs in our center and hence continued as part of the expert group. Two hundred seventy‐two (46.1%) of the procedures were performed by the expert group. The most common histology was mucosal adenocarcinoma (55.1%). Despite experts having a significantly higher number of difficult lesions (i.e. larger, fibrotic, and/or difficult position), they achieved higher dissection speed (10.3 vs. 6.7 cm^2^/h). This difference was statistically significant for both unadjusted and adjusted models (both having *P* < 0.0001). The en bloc (experts = 95.6%; trainees = 94.7%, *P* = 0.61) and R0 (experts = 85.7%; trainees = 83.6%, *P* = 0.50) resection rates were similar for both groups. Although nonexperts damaged more of the muscularis propria (18.6 *vs* 12.5%, *P* = 0.04), this did not translate into a significant difference in perforation (experts = 3.7%; trainees = 6.9%, *P* = 0.09) or delayed bleeding (experts = 2.9%; trainees = 4.4%, *P* = 0.34). Curative resection was not different between the groups (experts = 81.3%; trainees = 81.1%, *P* = 0.95). Efficacy and safety variables have been summarized in Table 2.

**Table 2:** Endoscopic submucosal dissection (ESD) outcomes per group model 2

	Experts	ESD trainees	*P*‐value
Procedure speed in cm^2^/h, mean (SD)*	10.3 (13.1)	6.7 (7.6)	<0.001
Procedure time in minutes, mean (SD)*	98.8 (73.0)	119.9 (71.1)	<0.001
Specimen area in cm^2^, mean (SD)*	15.1 (16.8)	12.6 (10.7)	0.03
En‐bloc resection, *n* (%)	260 (95.6)	301 (94.7)	0.61
R0 resection, *n* (%)	233 (85.7)	266 (83.7)	0.50
Curative resection, *n* (%)	221 (81.3)	258 (81.1)	0.95
Beyond fold, *n* (%)*	171 (62.9)	165 (51.9)	<0.01
F2 fibrosis, *n* (%)*	54 (19.9)	39 (12.3)	0.01
Ileocecal valve or anus involvement, *n* (%)*	27 (9.9)	14 (4.4)	<0.01
Muscularis propria damaged, *n* (%)*	34 (12.5)	59 (18.6)	0.04
Fever (>37.5°C), *n* (%)	32 (11.8)	39 (12.3)	0.85
PECS, *n* (%)	19 (7.0)	23 (7.2)	0.93
Perforation, *n* (%)	10 (3.7)	22 (6.9)	0.09
Delayed bleeding, *n* (%)	8 (2.9)	14 (4.4)	0.34
Emergency operation, *n* (%)	0 (0.0)	2 (0.6)	0.19

* *P* < 0.05.

Adjusted log linear regression of procedure speed (in cm/h) *versus* expertise and relevant confounders.

PECS, post‐colorectal ESD coagulation syndrome.

**Figure 2:**
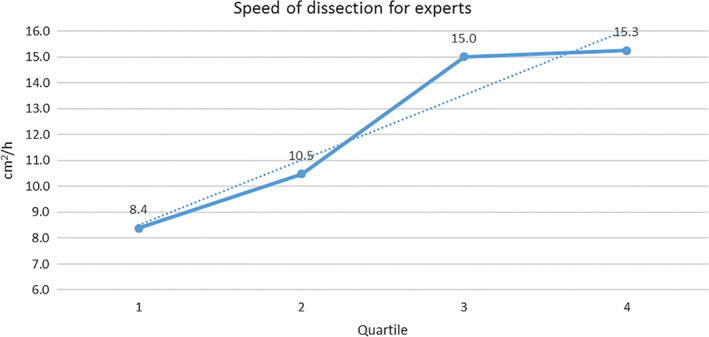
Endoscopic submucosal dissection speed evolution for expert endoscopists.

**Figure 3:**
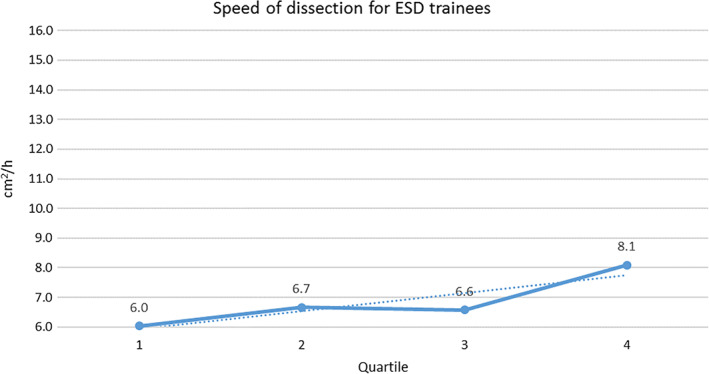
Endoscopic submucosal dissection (ESD) speed evolution for ESD trainees.

The dissection speed steadily increased with expertise. The trend of improvement in dissection speed is illustrated in Figure 2 (ESD dissection speed evolution for expert endoscopists) and Figure 3 (ESD dissection speed evolution for ESD trainees). In the adjusted log‐linear regression, eight variables were found to present statistically different results regarding average dissection speed: experience, fibrosis, curative resection, en bloc resection, free margins, lesion size, involvement of folds, and damage to muscularis propria, which independently affected speed (*P* < 0.05). ESDs performed by the expert group, with F0 fibrosis, with curative and en bloc resections, with free margins and without MP damage led to a higher average dissection speed. Interestingly, larger size and lesion over the fold were also associated with higher speed.

The data on average speed of dissection were divided into quartiles for better understanding of the evolution of each group. The quartiles were based on the total number of procedures performed by each group (ESD trainees and experts) divided by 4. For instance, all ESD trainees were allocated into Quartile 1 up to their fourth ESD, when they then passed through to Quartile 2. ESD experts were allocated into Quartile 1 up to their 25th ESD, when they then passed through to Quartile 2.

## Discussion

In our study, we have arbitrarily adopted 16 as the number of colorectal ESDs performed to be allocated into the “expert group.” This was considered sufficient based on previous ESD experience of the trainees (20–50 ESDs) and on the average number of colorectal ESDs performed by the trainees during their colorectal ESD training. Although the number of procedures to achieve proficiency is variable in the literature, our data suggest that a few dozen allow a safe and effective ESD. Moreover, this could be achieved even though it is performed at a slower pace. Hotta *et al*. studied 120 lesions and found a minimum number of 40 ESDs to avoid perforation and 80 to reach R0 rates similar to experts. This study was based on the data from a single expert endoscopist in Japan.^14^ A single Western operator with a similar background (i.e. hundreds of EMRs and few gastric ESDs) have shown a higher number of ESDs required for reaching the improvement plateau. After 152 procedures, the en bloc (R0) resection rate achieved was 92.4%. The speed of dissection has reached the 9 cm^2^/h threshold with 76 cases.^15^ Another single‐operator large cohort has found that although it was possible to reach expert‐level dissection speed and en bloc resection rates after over 300 colorectal ESDs, it was not possible to achieve the R0 nor complication rates expected for an expert.^16^ On the other hand, another single‐operator study from Germany has found numbers close to the expert standards with only 30 unsupervised cases.^17^ These studies illustrate the immense variability found, which is likely to be associated with endoscopist‐related factors. Hence a comprehensive study on learning curves for multiple endoscopists is important to accurately evaluate ESD training and achievement of expertise, mitigating the bias of individual particularities.

Some factors might influence the dissection speed such as fibrosis, difficult locations, and lesion size. They were taken into account in a log‐linear adjusted model and it was confirmed that even controlling for these factors, experts achieved higher dissection speed compared with ESD trainees. As endoscopists' expertise in ESD increases, so does the complexity of cases (e.g. larger lesions and more difficult locations) and the dissection speed. These may bias the outcomes toward a worse complication rate for experts.^18^ However, we have found that although the complexity and dissection speed were indeed higher for experts, the complications were not. Looking at the dissection speed graph evolution throughout the first and second half of procedures for ESD trainees and experts, it is possible to visualize a trend of continuity in learning and evolution through time.

Being one of the most complex procedures in gastrointestinal endoscopy, ESD comes with relative high risk of complications such as bleeding and perforation. Although the perforation and bleeding rates were numerically higher in the ESD trainee group, they were not statistically different from the expert group. Therefore, ESDs were safely performed and with similar efficacy (i.e. R0 and en bloc resection rates) by both ESD trainees and experts. This might sound unusual if only the number during the colorectal ESD training is considered. However, ESD during training is always supervised by an expert and all of our colorectal ESD trainees had previous experience with ESD. A recent meta‐analysis with 97 studies on ESDs found an overall perforation rate of 5.2% and a delayed bleeding rate of 2.7%. These rates varied depending on where the study took place. On the one hand, the pool of Asian studies had perforation and bleeding rates of 4.5 and 2.4%. On the other hand, the sum of non‐Asian studies had perforation and bleeding rates of 8.6 and 4.2%.^19^ Our study has shown a rate of perforation and delayed bleeding closer to other Asian studies.

In this study, the average dissection speed steadily increased over time for both ESD trainee group and expert group. Interestingly, even with a slower dissection speed than recommended (or because a slower and more cautious dissection was utilized), serious complications were not statistically different compared to experts. The fact that all ESDs performed by ESD trainees were supervised certainly contributed to this outcome. Nevertheless, knowing that ESD trainees can perform as safely and as efficaciously compared experts (when supervised) might be an important information for training centers. In addition, conversely to good outcomes regarding curative resection and complications, the dissection speed was always lower than the recommended 9 cm^2^/h for ESD trainees. This suggests adequate safety and efficacy outcomes might not be intertwined with dissection speed of 9 cm^2^/h or higher.

The limitations of this study include the low threshold for being considered an expert (only 16 colorectal ESDs) and the fact that all ESDs performed by the trainees were always supervised by an expert. Although only 16 colorectal ESDs were considered as a threshold for this study for logistic purposes, the results suggest that this number might be sufficient. Endoscopists selected for the colorectal ESD training program at Nagoya University Hospital must have prior experience with ESD in animal models and/or humans (between 20 and 50 ESDs).

In our center, all colorectal ESDs performed by the trainees are supervised by experts. It is expected that when supervised, the complication rates should be lower than when ESDs are performed without supervision. Therefore, our results might reflect only the early learning phase performance when this is done under supervision. However, as it is advised that initially ESDs should always be performed under supervision, our results are likely to be applicable to most cases of early learning curve for colorectal ESD. In addition, all our ESD experts have originated from following‐up ESD trainees. Therefore, it is possible to say that even after finishing the training and not being under supervision, “early ESD experts” were capable of maintaining/improving their dissection speed, efficacy, and safety when performing colorectal ESDs.

In conclusion, although dissection speed for colorectal ESD was significantly higher for experts, ESDs could be safely and efficaciously performed by ESD trainees.
